# A live-cell, high-content imaging survey of 206 endogenous factors across five stress conditions reveals context-dependent survival effects in mouse primary beta cells

**DOI:** 10.1007/s00125-015-3552-5

**Published:** 2015-03-14

**Authors:** Yu Hsuan Carol Yang, Quin F. Wills, James D. Johnson

**Affiliations:** 1 Department of Cellular and Physiological Sciences, Faculty of Medicine, Diabetes Research Group, Life Sciences Institute, University of British Columbia, 5358-2350 Health Sciences Mall, Vancouver, BC Canada V6T 1Z3; 2 Wellcome Trust Centre for Human Genetics, Weatherall Institute of Molecular Medicine, University of Oxford, Oxford, UK; 3 Weatherall Institute of Molecular Medicine, University of Oxford, Oxford, UK

**Keywords:** Autocrine/paracrine signalling, Beta cell apoptosis, ER stress, High-throughput screening, Islets

## Abstract

**Aims/hypothesis:**

Beta cell death is a hallmark of diabetes. It is not known whether specific cellular stresses associated with type 1 or type 2 diabetes require specific factors to protect pancreatic beta cells. No systematic comparison of endogenous soluble factors in the context of multiple pro-apoptotic conditions has been published.

**Methods:**

Primary mouse islet cells were cultured in conditions mimicking five type 1 or type 2 diabetes-related stresses: basal 5 mmol/l glucose, cytokine cocktail (25 ng/ml TNF-α, 10 ng/ml IL-1β, 10 ng/ml IFN-γ), 1 μmol/l thapsigargin, 1.5 mmol/l palmitate and 20 mmol/l glucose (all in the absence of serum). We surveyed the effects of a library of 206 endogenous factors (selected based on islet expression of their receptors) on islet cell survival through multi-parameter, live-cell imaging.

**Results:**

Our survey pointed to survival factors exhibiting generalised protective effects across conditions meant to model different types of diabetes and stages of the diseases. For example, our survey and follow-up experiments suggested that OLFM1 is a novel protective factor for mouse and human beta cells across multiple conditions. Most strikingly, we also found specific protective survival factors for each model stress condition. For example, semaphorin4A (SEMA4A) was toxic to islet cells in the serum-free baseline and serum-free 20 mmol/l glucose conditions, but protective in the context of lipotoxicity. Rank product testing supported the consistency of our observations.

**Conclusions/interpretation:**

Collectively, our survey reveals previously unidentified islet cell survival factors and suggest their potential utility in individualised medicine.

**Electronic supplementary material:**

The online version of this article (doi:10.1007/s00125-015-3552-5) contains peer-reviewed but unedited supplementary material, which is available to authorised users.

## Introduction

The loss of functional beta cell mass is critical in diabetes pathogenesis [1]. Research aimed at discovering beta cell survival factors has typically been conducted one at a time and has been limited by prior knowledge [2–4]. Glucagon-like peptide 1 (GLP1) is considered a gold standard for beta cell protective factors [5]. Although local GLP1 increases islet transplant success in animals [6], clinical evidence to support its efficacy to durably increase human beta cell mass is lacking. Clearly, there is an unmet need to identify more and better beta cell survival factors.

Our efforts to identify novel factors with sustained anti-apoptotic effects led us to mine expression databases and characterise locally acting pro-survival factors in the islet secretome, which includes >200 expressed ligands [7]. Our initial analyses of candidates revealed glucose-dependent protective roles for Notch, Netrin and Slit [7–9]. However, without a side-by-side comparison, it is impossible to determine the relative merits of each candidate. We recently developed high-throughput, kinetic, live-cell imaging methods that allow the effects of hundreds of factors on multiple cell death parameters to be simultaneously evaluated in dispersed primary islet cells over relatively long periods of time in culture [10].

Here, we surveyed 206 factors, rationally chosen based on previous bioinformatic analysis [7], and ranked their effects on islet cell survival under five distinct stress conditions. We found many factors with previously unreported pro-survival effects. Remarkably, each stress condition was associated with a relatively distinct set of protective and deleterious factors, consistent with fundamental mechanistic differences in the cell death pathways. This first systems level analysis has important implications for the development of beta cell protective and regenerative therapies [11].

## Methods

### Multi-parameter imaging

Mice were housed in accordance with the University of British Columbia Animal Care Committee guidelines. After overnight culture, isolated islets were dispersed and cultured for 48 h in 384-well plates. After serum-free medium washes, cells were stained with Hoechst 33342, propidium iodide (PI), and annexinV-conjugated-AlexaFluor647 prior to imaging with ImageXpress^MICRO^ (Molecular Devices, Sunnyvale, CA, USA) [9, 10, 12]. Cells treated with the following conditions (all serum-free): 5 mmol/l glucose; 20 mmol/l glucose; 25 ng/ml TNF-α, 10 ng/ml IL-1β and 10 ng/ml IFN-γ cocktail; 1 μM thapsigargin; 1.5 mmol/l palmitate (complexed to BSA at 6:1 molar ratio) [10, 13], were imaged every 3 h for 60 h in the presence of 10% vol./vol. FBS (positive control) or test factors (Electronic Supplementary Material [ESM] Table 1) at 10 nmol/l final concentration. Our imaging protocols have been described [9, 10, 12] (see ESM Methods for further details).

### Data analysis

Images were analysed using MetaXpress software (Molecular Devices) [9, 10, 12]. Cell loss, PI^+^ and AnnexinV^+^PI^-^ cells were calculated, as was AUC between 0–24 h and 24–48 h. For each individual experiment, *z* scores were determined based on (*x* − median)/MAD, where MAD represents the median absolute deviation. Rank product testing was used to identify factors with consistent effects across time and between replicate experiments and the results are presented in principal component analysis (PCA) plots, wherein the first component in the PCA plot illustrates the agreement between the days, whereas the second component highlights their differences. For western blot studies, analysis of variance was calculated using GraphPad Prism (San Diego, CA, USA), and results were considered statistically significant when *p* < 0.05, using the Tukey–Kramer post-hoc test (see ESM Methods for further details). Data are expressed as mean ± SEM, unless otherwise stated.

## Results

### Factors with generalised effects on islet cell survival across multiple conditions

**Fig. 1:**
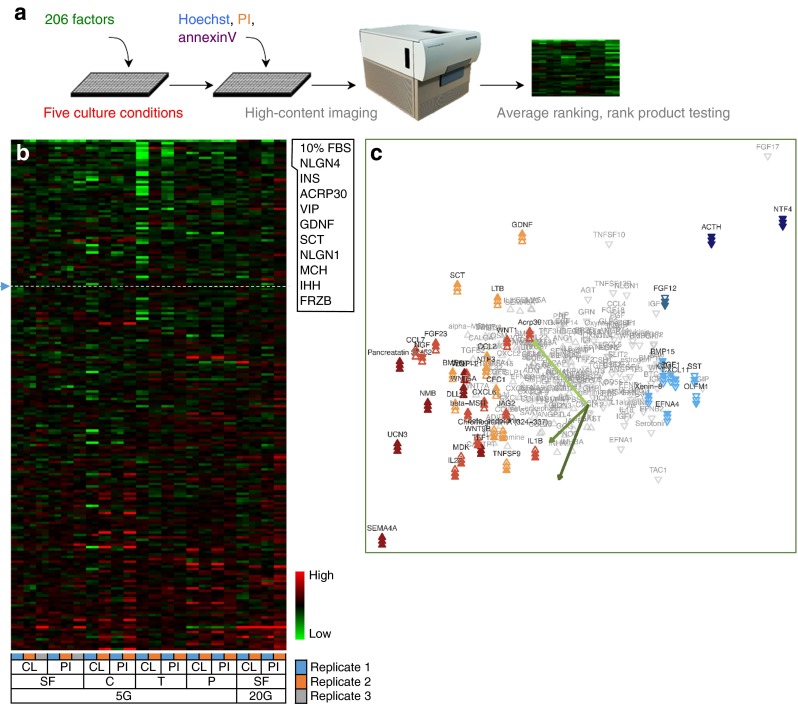
Effects of 206 factors on islet cell survival across five conditions. (**a**) Study design. (**b**) PI-positive (PI^+^) cell percentage was determined following treatments with 206 factors [10 nmol/l] in one of five conditions. Data presented as *z* scores for the 0–24 and 24–48 h time intervals for each replicate. Factors were ranked for overall protective effect (low levels of cell loss [CL] and low levels of PI^+^ cells [PI] equates to high protection). SF, serum-free; C, cytokines; T, thapsigargin; P, palmitate. The top ten factors are listed to the side. (**c**) Rank product analysis of PI^+^ cell data (see ESM Methods for details). The first component in the PCA plot illustrates the agreement between the days, whereas the second component highlights their differences. Nominally significant factors for any one day are highlighted with three arrows (representing 3 days: top arrow, day 1; middle arrow, day 2; and bottom arrow, day 3). Coloured arrows are significant for that day. See ESM Table 1 for protein abbreviations

We set out to compare endogenous soluble factors that may directly promote or inhibit survival under five controlled stress conditions in dispersed mouse islet cells. Mouse islet cells were chosen because of their reproducibility and low baseline rates of apoptosis, relative to human islet cells where the in vitro rates of cell death are typically much higher and more variable. The cells were concurrently treated with a library of 206 recombinant factors compiled using our previous bioinformatics analysis of islet cell ligands and receptors [7], along with candidates from the literature (Fig. 1a). Row and column variability in cell seeding density and cell death was negligible (ESM Fig. 1a,b). Cells cultured in 0.1% FBS and in the absence of serum displayed the same levels of death (ESM Fig. 1c). Analysis of dispersed mouse insulin-1 promoter-green fluorescent protein (MIP-GFP) islet cells suggested that beta cells were more sensitive to these stress conditions compared with the non-beta cell population within the same cultures (ESM Fig. 1c,d).

**Fig. 2:**
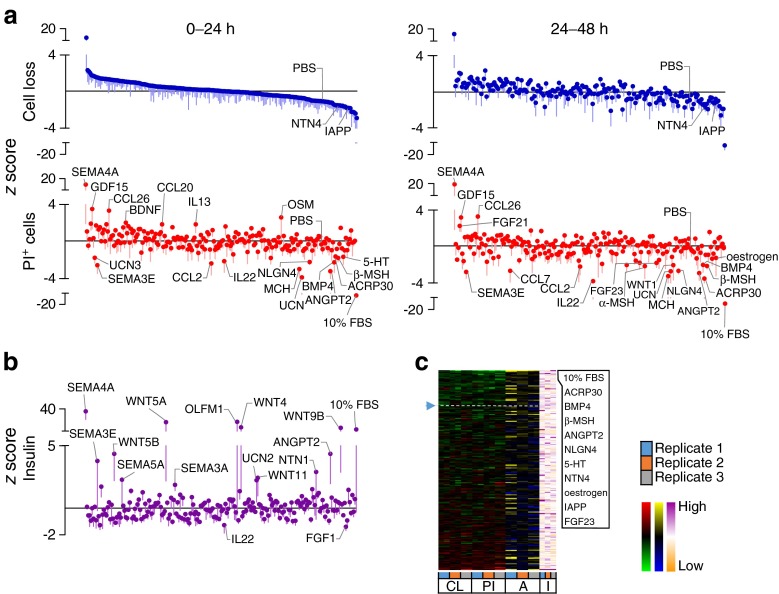
Effects of 206 factors in a baseline serum-starved condition. Islet cells were treated with 206 factors in 5 mmol/l glucose serum-free conditions. (**a**) Cell loss and PI-positive cell percentage (PI^+^). 10% FBS was a positive control. *z* scores for 0–24 and 24–48 h time intervals after ordering based on the level of cell loss in 0–24 h (*n* = 3, mean ± SEM). Solid line represents median. (**b**) Insulin in the culture media collected after 72 h (*n* = 3, mean ± SEM). (**c**) Factors ranked based on low cell loss (CL) and low PI^+^ (PI) cell number (green-red heat maps). AnnexinV^+^PI^-^ cells (A) are shown with blue-yellow and insulin (I) with orange-purple. The top ten protective factors under each condition are listed. See ESM Fig. 8 for PCA plot of rank product testing analysis; see ESM Table 1 for protein abbreviations

**Fig. 3:**
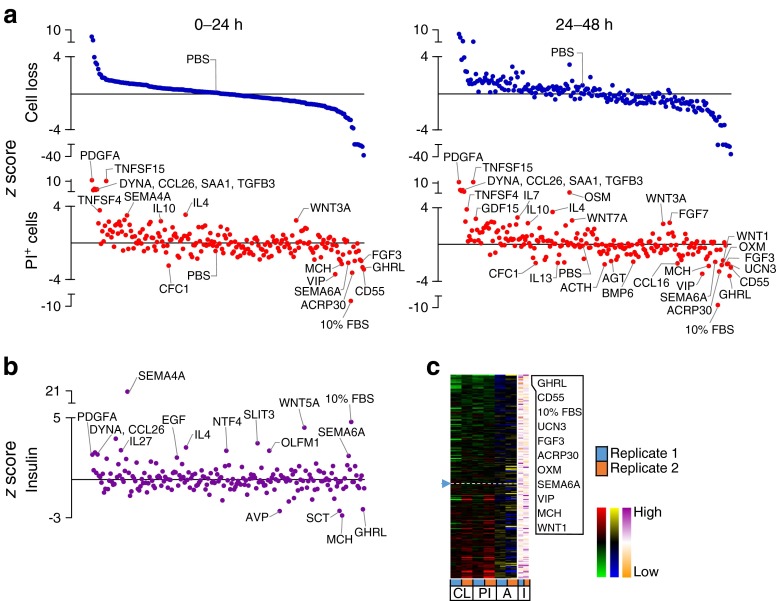
Effects of 206 factors in the context of toxic cytokines. Cells treated with 206 factors and cytotoxic cytokines in 5 mmol/l glucose serum-free conditions. (**a**–**c**) Data presented as in Fig. 2 (*n* = 2, mean). See ESM Fig. 9 for PCA plot of rank product testing analysis; see ESM Table 1 for protein abbreviations; DYNA, dynorphin A

**Fig. 4:**
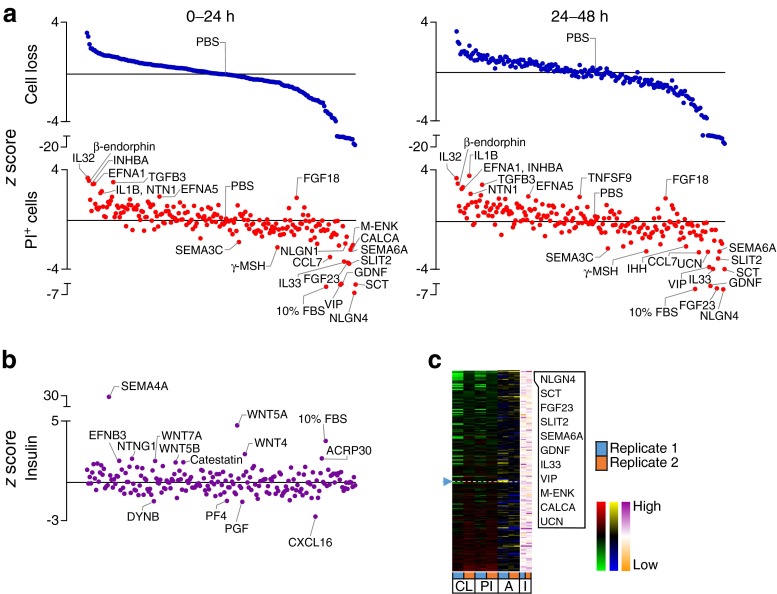
Effects of 206 factors in the context of ER stress. Cells treated with 206 factors and thapsigargin in 5 mmol/l glucose serum-free conditions. (**a**–**c**) Data presented as in Fig. 2 (*n* = 2, mean). See ESM Fig. 10 for PCA plot of rank product testing analysis; see ESM Table 1 for protein abbreviations; DYNB, dynorphin B

**Fig. 5:**
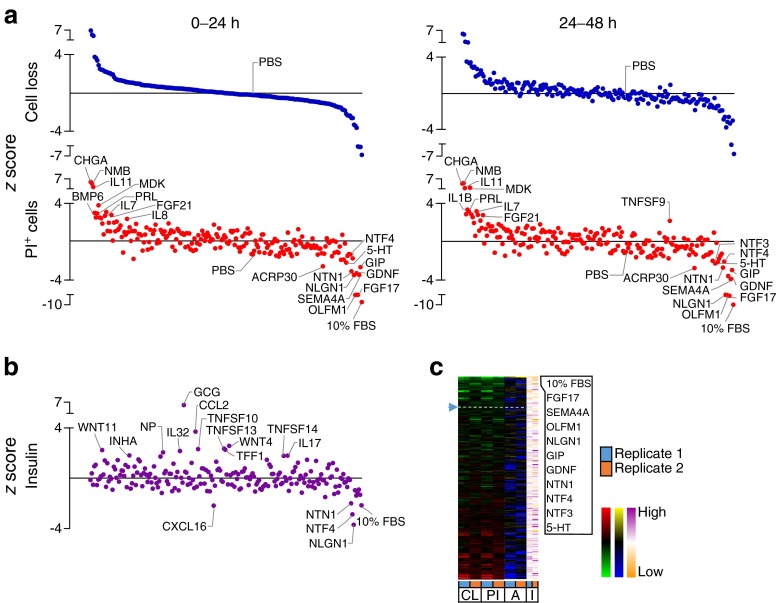
Effects of 206 factors in the context of lipotoxicity. Cells treated with 206 factors and palmitate in 5 mmol/l glucose serum-free conditions. (**a–c**) Data presented as in Fig. 2 (*n* = 2, mean). See ESM Fig. 11 for PCA plot of rank product testing analysis; see ESM Table 1 for protein abbreviations

**Fig. 6:**
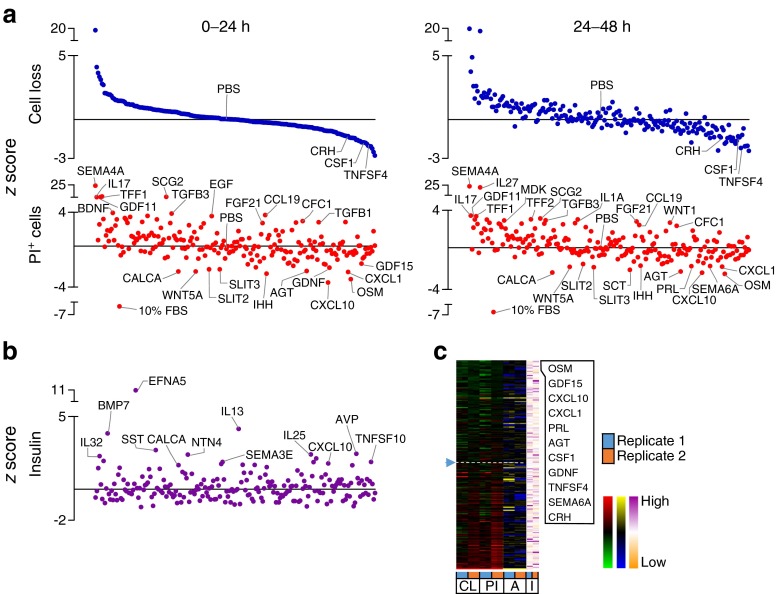
Effects of 206 factors in the context of hyperglycaemia. Cells treated with 206 factors in 20 mmol/l glucose serum-free conditions. (**a**–**c**) Data presented as in Fig. 2 (*n* = 2, mean). See ESM Fig. 12 for PCA plot of rank product testing analysis; see ESM Table 1 for protein abbreviations

High-content, image-based analysis can simultaneously assess multiple parameters for internal validation, providing a level of redundancy that increases confidence in the results. We measured the loss of Hoechst-positive cells and the accumulation of PI-positive cells as indices of cell death. This captures multiple forms of cell death, including the ‘partial apoptosis’ that we recently demonstrated is the predominant mode of death in cultured primary beta cells [10]. The results of these studies revealed both pro-survival and pro-death factors within our library of endogenous ‘biologic’ factors, consisting mostly of recombinant full-length proteins (Figs 2, 3, 4, 5 and 6). Heatmaps showed the relative agreement between the replicate experiments (Panel c in Figs 1, 2, 3, 4, 5 and 6, ESM Table 2). As expected, cells treated with our positive control (10% FBS, presumably containing high concentrations of many islet survival factors) displayed the lowest PI levels. The agreement between the measurements of cell loss and PI incorporation can be seen in the distribution of the top and bottom parts of Panel a in Figs 2, 3, 4, 5 and 6, and is especially evident in stress conditions with larger effects (i.e. cytokines, thapsigargin, palmitate). Divergence is expected if specific factors modified the adhesion of dead or dying cells. The number of early apoptotic, AnnexinV-positive and PI-negative cells was also analysed, but as we have recently reported, these are relatively rare and their analysis is less informative [10].

First, we compared effects of each factor across all of the tested stresses to identify factors that might be generally protective or generally deleterious. We present the data here, and elsewhere, in multiple ways. The heat map in Fig. 1b represents the simple average rankings of PI^+^ cell count and cell loss, at two time blocks (0–24 h, 24–48 h), across all conditions (see ESM Methods). This analysis is meant to be exploratory and does not provide statistical information on the extent of protection or relative differences in protection among factors. Nevertheless, several known anti-apoptotic factors, including insulin [4, 14, 15] and adiponectin [16–19], were highlighted, as well as unexpected factors. Pan-protective factors included those that were ranked amongst the top ten most potent survival factors under one or more conditions, along with those displaying moderate but consistent effects across all conditions. A sortable list of these results can be found in ESM Table 2. We validated the survival effects of olfactomedin 1 (OLFM1), across multiple conditions, and other factors on MIP-GFP beta cells (ESM Fig. 2-5). We confirmed that OLFM1 dose-dependently promotes human islet cell survival under lipotoxic conditions (ESM Fig. 6). Mechanistically, OLFM1 significantly downregulated p38 MAPK and caspase-3-dependent apoptotic signalling (*p* < 0.05; ESM Fig. 7).

We measured the consistency between replicates using rank product testing. A normalised robust ‘cell death score’ was log_2_ scaled and then divided by the MAD of scores for the plate. Results for each well were then aggregated for day 1, day 2 and day 3 as the median cell death score for the day, providing a measure of the durability of protection. Statistical significance was measured as the reproducibility of cell death score ranks across replicate plates. As the factors were pre-selected, with expected and non-independent effects, we reasoned that naive multiple testing correction would be too conservative. Factors that were nominally significant (i.e. *p* < 0.05) on any day are illustrated with a triple triangle, with the significant days illustrated by filled triangles in PCA plots (Fig. 1c, ESM Fig. 8-12). The protective effect of 10% serum, our positive control, was globally statistically significant (*p* < 0.05) over all 3 days of testing, but is not shown in PCA plots so that the test factors can be more easily viewed. The complete analysis, with *p* values for each factor can be found in ESM Table 3. Semaphorin 4A (SEMA4A) held up against the most conservative of multiple testing correction, and 41 nominally statistically significant factors were identified as being pro- or anti-survival over all 3 days tested. Each will require further testing to rule out multiple testing false positives. Using this approach of nominal statistical significance, adrenocorticotropic hormone (ACTH) and neurotrophin 4 were consistently significantly protective over all 3 days (*p* < 0.05), whereas OLFM1, somatostatin, chemokine (C-X-C motif) ligand 11 (CXCL11), fibroblast growth factor (FGF)1, IGF1, bone morphogenic protein 15, ephrin 4A, xenin 8 and FGF12 showed significant time-dependency of protection. The results differ from the raw average of rankings for several reasons, including the omission of cell count data (which was informative in the qualitative analysis, but difficult to combine into the ‘cell death score’), the adoption of raw data normalisation and the inclusion of the 3rd time block (49–60 h). Collectively, results of both qualitative and statistical analyses suggest that factors exist that may protect islet cells under a wide range of stress conditions.

### Stress-specific factors affecting islet cell survival

Comparing the effects of our chosen factors among stresses revealed that each stress had a relatively distinct complement of pro-survival factors and that there was little overlap between the highly ranked and nominally significant pro-survival or anti-survival factors found for each of the five stress conditions (Figs 1, 2, 3, 4, 5 and 6). For example, oncostatin M, from the cytokine family that includes leukaemia inhibitory factor, granulocyte-colony stimulating factor and IL-6 [20], was protective in context of both thapsigargin and high glucose, but no such evidence could be found in the context of low glucose serum withdrawal and the toxic cytokine cocktail (Fig. 6, ESM Table 2). Members of the neuroligin family, including NLGN1 and NLGN4, showed bias towards protection in endoplasmic reticulum (ER) stress and lipotoxicity, which we and others have shown are mechanistically linked [13, 21], and these findings were confirmed with secondary validation (ESM Fig. 4 and 5).

Comparing different stress conditions also revealed factors that were pro-survival under one condition, but pro-death under others. Most strikingly, semaphorin 4A, known for its roles in axon guidance, morphogenesis, carcinogenesis and immunomodulation [22], was the 2nd highest ranked (and most statistically consistently highly ranked) protective factor in the context of palmitate lipotoxicity, but it promoted cell death under all other conditions tested, including the baseline serum-free condition and the high glucose serum-free condition where it was the most consistently toxic factor (*p* < 0.05; Figs 1, 2, 3, 4, 5 and 6).

Some of our stress conditions were more resistant to protection than others (Figs 1, 2, 3, 4, 5 and 6). For example, only 37 factors provided any numerical protection of islet cells from palmitate above the negative PBS control, with the rest of the factors being apparently neutral or exacerbating lipotoxic cell death (Fig. 5). On the other hand, more than half of the factors provided some protection in the context of ER stress induced by a moderate dose of thapsigargin (Fig. 4). The shallow shape of the curve in Fig. 2a suggests that serum withdrawal alone was not a very severe stress in our studies, most likely because the islet cells can supply themselves with autocrine/paracrine survival factors.

### Concentration and time dependence of beta cell survival factors

We compared the concentration-dependent effects of each factor on cell survival under 5 mmol/l serum-free conditions (ESM Fig. 13). Some factors showed classical concentration-dependent effects. However, other factors exhibited bell-shaped dose–response curves, a phenomenon we have consistently observed with insulin [4, 14]. Temporal analysis revealed that some factors showed protective effects throughout the entire time course, while others were only protective early. It remains to be determined whether these transiently effective factors were rapidly degraded or taken up following treatment or whether the cells displayed receptor desensitisation.

When survival effects were analysed by pooling rank data from both the 0.1 nmol/l and 10 nmol/l concentrations, some factors that were not originally identified as protective when given in moderate concentrations, were shown to have efficacy at the lower concentrations (ESM Fig. 14). For example, cryptic family 1, γ-melanocyte stimulating hormone, somatostatin and ephrin-B2 were ranked with higher protective effects when the low and moderate concentrations were taken into consideration. Rank product testing of the entire 0.1 nmol/l data set (i.e. all factors across all tested conditions) identified hepatocyte growth factor, vascular endothelial growth factor-C, sonic hedgehog and serum amyloid-A1 as factors with statistically significant consistent protection across all 3 days at this dose (*p* < 0.05; ESM Fig. 15). Identifying factors with pro-survival effects under lower concentrations is widely considered to be important for therapeutic development because low effective concentrations may help reduce off-target effects.

### Classification of survival factors by signal transduction pathways

**Fig. 7:**
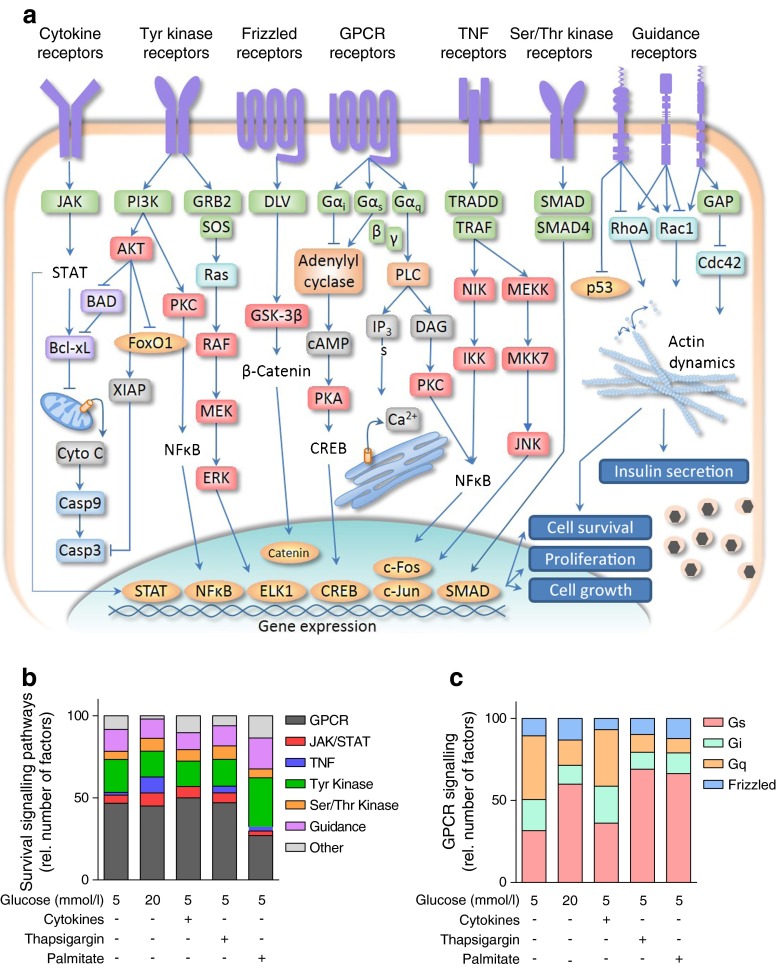
Manual annotation of signal transduction pathways of islet cell survival factors. (**a**) Canonical signalling pathways regulated by top factors. (**b**) The proportion of factors displaying protective effects with *z* scores values below 2× MAD of each condition (for cell loss or PI^+^ measurements in the 0–24 or 24–48 h intervals) categorised by signalling pathway. (**c**) Proportion of factors signalling through specific G-protein-coupled receptor (GPCR) pathways; rel., relative; see ESM Table 5 for protein abbreviations

Qualitative analysis of the canonical signalling pathways stimulated by the protective factors revealed that pro-survival signalling could be mediated by a number of known pathways, including the janus kinase (JAK)–signal transducer and activator of transcription (STAT) cytokine receptors, G-protein-coupled receptors, tyrosine kinase receptors, serine/threonine kinase receptors and axon guidance receptors (Fig. 7a, b). These analyses revealed that specific signalling pathways were more important in the context of certain stresses, relative to others. In the baseline serum withdrawal condition, factors that stimulate phospholipase C and/or the activation of adenylyl cyclase tended to be protective, whereas factors that inhibit adenylyl cyclase were less effective. In the context of thapsigargin or palmitate, activation of adenylyl cyclase was identified as the predominant pro-survival G-protein-mediated pathway.

### Context-dependent effects on chronic insulin release

We also assessed insulin accumulation in the media. We identified factors that promoted both survival and insulin accumulation in the media, including angiopoietin 2 in the 5 mmol/l glucose serum-free condition (Fig. 2b). Our crude data do not allow us to distinguish whether the survival effects were due to insulin independent survival signalling or pro-survival autocrine insulin signalling [4, 15]. Other factors were protective while inhibiting insulin accumulation in the media. It is known that inhibition of calcium flux can protect beta cells under specific conditions [23, 24], while these same manipulations block insulin secretion [25]. We also detected factors, such as semaphorin 4A, which triggered cell death and increased insulin in media, likely secondary to the loss of cell integrity (Figs 2b and 3b). The effects of soluble factors on insulin release were context-dependent. Analysis of insulin release was not a primary endpoint in the present study, although these data nonetheless provide a starting point for additional detailed studies.

## Discussion

The goal of the present study was to simultaneously compare the effects of 206 putative beta cell survival factors, under multiple conditions, using a newly developed imaging platform. Our survey pointed to many previously unappreciated factors that may protect islet cells. A principal observation was that each cellular stressor examined appears to require its own unique set of protective factors, and that factors (i.e. SEMA4A) can switch from pro-death to pro-survival. These findings have significant implications for the understanding of the molecular mechanisms controlling beta cell fate and for the development of therapeutic approaches to prevent or treat type 1 or type 2 diabetes at various disease stages.

Factors were selected for testing based on their expression or the expression of their receptors in islets. Harnessing local islet autocrine and/or paracrine survival factor signalling may be an ideal scenario for diabetes prevention or treatment. Many local factors act on self-limiting signalling mechanisms that prevent over-stimulation. Insulin, for example, is a potent and self-limiting islet survival factor and physiological doses of insulin can increase beta cell proliferation [15, 26, 27]. An unbiased search for other potent survival factors not involved in peripheral metabolism is needed. Another approach would be to exploit pancreatic development factors, including Notch, TGF-beta superfamily, FGFs and bone morphogenic proteins [8, 28–31]. We also cannot overlook the potential importance of distally secreted factors, including adipokines such as adiponectin.

In type 1 diabetes, beta cells may be destroyed by a combination of toxic cytokines and other factors including granzyme, perforin and Fas [32, 33]. Studies have also implicated ER stress in beta cell death associated with type 1 diabetes [34]. Thus, it is possible that factors showing protection in both of these conditions may be therapeutic in type 1 diabetes. In type 2 diabetes, excessive fatty acids and ER stress act through partially common pathways to increase beta cell death following the initial compensation phase [13, 35, 36]. These stresses had in common protection by OLFM1, neuroligin family members and members of the FGF family. Notably, the known beta cell anti-apoptotic factors, insulin and IGF2 [14, 37], were protective in the context of lipotoxicity more consistently relative to the other stresses. Persistent hyperglycaemia, present in poorly controlled type 1 or type 2 diabetes, induces further beta cell apoptosis [38–40] and factors that are protective under this condition may be candidates for adjunct or second-line treatments.

Some factors promoted survival under only one condition. The most striking of these was the palmitate specific pro-survival effect of semaphorin 4A, which acts through plexin receptors expressed in beta cells [41]. Clearly, this factor would not be an ideal therapeutic target owing the presence of multiple, concurrent beta cells stresses in vivo. While it might seem counter-intuitive to some that islets would respond to an endogenous factor with significant death, it is possible that the local concentrations of semaphorin 4A are lower than the toxic levels employed in our experiments and that this factor plays an important role in constraining excessive beta cell growth. We expect that functional beta cell mass is controlled in vivo by a robust balance of positive and negative factors [31].

In addition to the discovery of stress-specific islet cell survival factors, our analyses also enabled the identification of factors with generalisable survival effects across different conditions. Using the simple average ranking analysis of both PI and cell loss data over the first two days, the most broadly effective protective factors appeared to be melanin-concentrating hormone, vasoactive intestinal peptide (VIP) and adiponectin. Melanin-concentrating hormone plays a role in obesity and has been implicated in islet growth [42]. VIP has known effects on insulin secretion [43]. Adiponectin is an insulin sensitising adipokine that protects beta cells against multiple stresses as found in our research and that of others [16–19]. Anti-apoptotic effects of adiponectin may not extend to all cell types [44], suggesting a degree of beta cell specificity. Using the rank product testing to assess the consistency of PI incorporation observations over 3 days, we identified several ‘pan-protective’ factors including neurotrophin 4, ACTH, FGF 12, somatostatin and OLFM1. We believe both the simple average ranking and the rank product testing have value, as they represent different aspects of the data and may be differentially influenced by multiple factors, including peptide stability in storage over the ~3 years these studies took place.

Our findings complement previous studies on the pro-survival signalling mediated by axon guidance factors, netrins and slits [7, 9]. We observed effects of slits, neuroligins and semaphorins in our parallel comparisons, all factors known to modulate the actin cytoskeleton and play roles in pancreas morphogenesis [30]. In the present study, we chose to pursue OLFM1, also known as noelin 1 or pancortin. OLFM1 is a modulator of Wnt signalling involved in neuronal development and axon elongation [45], which interacts with the Nogo A receptor (NgR1) complex [46] expressed in beta cells [41]. Our studies demonstrated a dose-dependent effect of OLFM1 on mouse and human beta cell survival in a number of conditions. Thus, despite a lack of adequate statistical power within each condition, our survey/ranking approach identified a novel beta cell survival factor, with conserved effects in mouse and human cells. Although confirming the effects of other interesting factors in human islets is beyond the scope of this project, it is essential to take most of these observations beyond the exploratory stage. We expect our survey will be broadly applicable to human islets, because the factors we surveyed were selected based on both rodent and human expression studies [7] and because our previous studies suggest broadly similar cell survival pathways in mouse and human islets [7, 8, 13, 47]. Recent RNA sequencing analysis confirms remarkable similarity between species, with only ~1.5% of genes being unique to either mouse or human beta cells [41, 48]. However, approximately 6% of genes show species enrichment, including 61 ligands or receptors (ESM Table 4), including the enrichment of *Prlr*, *Ghr* and *Cntfr* in mouse beta cells compared with human beta cells [41, 48]. Interestingly, SEMA4A is also enriched in mouse beta cells [41, 48]. One caveat is that the mice used to generate these transcriptomic data sets were 3–6 months old (a time when mice are still growing), whereas the humans donors averaged ~55 years of age, meaning that some of the gene expression differences can be ascribed to relative age differences.

Collectively, our survey of endogenous soluble factors identified multiple hormones/cytokines/growth factors with robust islet cell survival effects under five stress conditions designed to model aspects of type 1 and type 2 diabetes. Perhaps the most important finding was that beta cells were best protected from each specific stress condition by a relatively distinct set of factors. This observation provides important insight into the complexity of beta cell survival signalling pathways and guides therapeutic efforts to protect beta cells.

## Electronic supplementary material

Below is the link to the electronic supplementary material.

ESM Methods (PDF 91 kb)

ESM Fig. 1 (PDF 341 kb)

ESM Fig. 2 (PDF 820 kb)

ESM Fig. 3 (PDF 815 kb)

ESM Fig. 4 (PDF 815 kb)

ESM Fig. 5 (PDF 805 kb)

ESM Fig. 6 (PDF 323 kb)

ESM Fig. 7 (PDF 466 kb)

ESM Fig. 8 (PDF 716 kb)

ESM Fig. 9 (PDF 806 kb)

ESM Fig. 10 (PDF 453 kb)

ESM Fig. 11 (PDF 296 kb)

ESM Fig. 12 (PDF 506 kb)

ESM Fig. 13 (PDF 651 kb)

ESM Fig. 14 (PDF 229 kb)

ESM Fig. 15 (PDF 288 kb)

ESM Table 1 (PDF 107 kb)

ESM Table 2 (XLSX 485 kb)

ESM Table 3 (XLSX 324 kb)

ESM Table 4 (XLSX 58 kb)

ESM Table 5 (XLSX 19 kb)

## Abbreviations

ACTH: Adrenocorticotropic hormone
ER: Endoplasmic reticulum
FGF: Fibroblast growth factor
GLP1: Glucagon-like peptide-1
JAK: Janus kinase
MAD: Median absolute deviation
MIP-GFP: Mouse insulin-1 promoter, green fluorescent protein
OLFM1: Olfactomedin 1
PCA: Principal component analysis
PI: Propidium iodide
SEMA4A: Semaphorin 4A
VIP: Vasoactive intestinal peptide
