# Supplementary material for: A live-cell, high-content imaging survey of 206 endogenous factors across five stress conditions reveals context-dependent survival effects in mouse primary beta cells
Source: Diabetologia. 2015 Mar 14;58(6):1239–49. doi: 10.1007/s00125-015-3552-5 (PMC4415993; doi:10.1007/s00125-015-3552-5)
Supplement: Supplementary file 1 — (PDF 91 kb) [file 125_2015_3552_MOESM1_ESM.pdf]

ESM Methods

Multi-parameter imaging

Pancreatic islets were isolated using collagenase and filtration from 20- to 30-week-old male C57BL/6J mice and MIP-GFP transgenic mice expressing EGFP under the control of a long fragment of the *Insulin 1* promoter [1](Jax, Bar Harbor, USA). Mice were housed in accordance with the University of British Columbia Animal Care Committee guidelines. Hand-picked islets were cultured overnight (37°C, 5% CO_2) in RPMI1640 medium (Invitrogen, Burlington, Canada) with 5 mmol/l glucose (Sigma, St. Louis, USA), 100 units/ml penicillin, 100 μg/ml streptomycin (Invitrogen) and 10% vol/vol FBS (Invitrogen) as described in more detail elsewhere [2].

For cell death assays, mouse islets were dispersed [2] and seeded into 384-well plates at 4000 cells/well. 48 hours following seeding, cells were washed with serum free RPMI medium, and stained with 60 ng/ml Hoechst 33342 (Invitrogen), 0.6 μg/ml propidium iodide (PI: Sigma), and 1:400 dilution of annexinV-conjugated-AlexaFluor647 (Invitrogen) for 1 hour prior to imaging of basal cell death with ImageXpress^{MICRO} (Molecular Devices, Sunnyvale, USA) at 37°C and 5% CO_2 [3]. Following treatments, cells were imaged at 3 hour intervals for 60 hr. Treatments included 5 and 20 mmol/l glucose serum free RPMI medium supplemented with a cytokine cocktail (25 ng/ml TNF-α, 10 ng/ml IL-1β, 10 ng/ml IFN-γ: R&D Systems, Minneapolis, USA), 1 μM thapsigargin, and/or 1.5 mmol/l palmitate (complexed to BSA at 6:1 molar ratio)[3, 4]. Serum depletion, achieved through sequential washing of the cells with serum free RPMI medium, mimicked mild nutrient deprivation and eliminated any potential synergistic effects with unknown factors in the serum. 10% vol/vol FBS was a positive control for unstressed cells. Factors were transferred with PerkinElmer Janus liquid handler (Waltham, Massachusetts, USA). To achieve a 10 nmol/l final concentration, aliquots of 3 μl of each factor at 300 nmol/l were pinned into 384-well plates and stored at -80°C. On the day of the start of the experiment, factors were thawed and 12 μl of the stress treatment medium was added to each well, yielding 60 nmol/l of each factor. Following imaging of the serum starved cells, 10 μl of each treatment was transferred into each well using the onboard ImageXpress^{MICRO} robotics, resulting in a final concentration of 10 nmol/l, unless otherwise indicated. Our established imaging protocols and analysis

routines have been previously described in more detail [3, 5]. Media were collected 72 hours following treatments and insulin in the media was assayed by radioimmunoassay (Rat insulin RIA kit, Millipore, Billerica, USA).

Data analysis

Following image analysis using MetaXpress software (Molecular Devices), cell loss was calculated relative to the amount of viable cells present in the time point prior to the treatments. The level of PI+ and AnnexinV+PI- cells were calculated relative to the total cell count in each time point. The accumulation of cell loss, PI+ cells, and AnnexinV+PI- cells was extracted from the area under the curve between 0-24 hours and 24-48 hours. *Z*-score values were determined for each individual experiment based on (x – median)/MAD, where MAD represents the median absolute deviation. Data are expressed as mean ± SEM unless otherwise indicated. Ranks were simply averaged across PI and cell loss values, and across 0-24 hour and 25-48 hour time blocks, to provide an indication of protection or anti-protection in a given condition. These ranks were averaged across all conditions to provide an indication of factors with effects on survival across multiple conditions.

In addition to the presentation of averaged ranks, we also performed rank product testing to identify factors with consistent effects across time and between replicate experiments. Prior to this analysis, data were normalized in the following way, so that the positive versus negative values would have biological significance. Cell counts (proportion of the median of first three measurements) and PI+ fluorescence (proportion of PI+ cells) were normalised to correct for unwanted fixed and random experimental effects. Per plate and per time point each well was divided by the mean value of PBS treated wells and log2 scaled for further robustness. Cell counts per plate or PI+ fluorescence per plate were also divided by the median absolute deviation of cells counts for the plate. Results for each well were then aggregated for day 1, day 2 and day 3 as the median value for the day. Statistical significance was measured as the reproducibility of ranks across replicate plates using rank product analysis (RankProd R library, 1 million permutations; Hong et al 2011. *RankProd: Rank Product method for identifying differentially expressed genes with application in meta-analysis*. R package version 2.38.0). Only PI+ data, not cell counts, were used in statistical calculations or principal component analysis (PCA) because the former is a more specific measure of cell death, whereas the later can include effects on proliferation and cell adhesion.

For Western blot studies, ANOVA was calculated using Prism software from GraphPad (La Jolla, CA, USA), and results were considered statistically significant when $p<0.05$, using the Tukey-Kramer post-hoc test.

References for Supplementary Research Design and Methods

[1] Hara M, Wang X, Kawamura T, et al. (2003) Transgenic mice with green fluorescent protein-labeled pancreatic beta -cells. Am J Physiol Endocrinol Metab 284: E177-183
[2] Yang YH, Szabat M, Bragagnini C, et al. (2011) Paracrine signalling loops in adult human and mouse pancreatic islets: netrins modulate beta cell apoptosis signalling via dependence receptors. Diabetologia 54: 828-842
[3] Yang YH, Johnson JD (2013) Multi-parameter single-cell kinetic analysis reveals multiple modes of cell death in primary pancreatic beta-cells. J Cell Sci 126: 4286-4295
[4] Jeffrey KD, Alejandro EU, Luciani DS, et al. (2008) Carboxypeptidase E mediates palmitate-induced beta-cell ER stress and apoptosis. Proc Natl Acad Sci U S A 105: 8452-8457
[5] Yang YH, Vilin YY, Roberge M, Kurata HT, Johnson JD (2014) Multi-parameter screening reveals a role for Na+ channels in cytokine-induced beta-cell death. Mol Endocrinol 126: 4286-4295
