# Supplementary material for: A live-cell, high-content imaging survey of 206 endogenous factors across five stress conditions reveals context-dependent survival effects in mouse primary beta cells
Source: Diabetologia. 2015 Mar 14;58(6):1239–49. doi: 10.1007/s00125-015-3552-5 (PMC4415993; doi:10.1007/s00125-015-3552-5)
Supplement: Supplementary file 2 — (PDF 341 kb) [file 125_2015_3552_MOESM2_ESM.pdf]

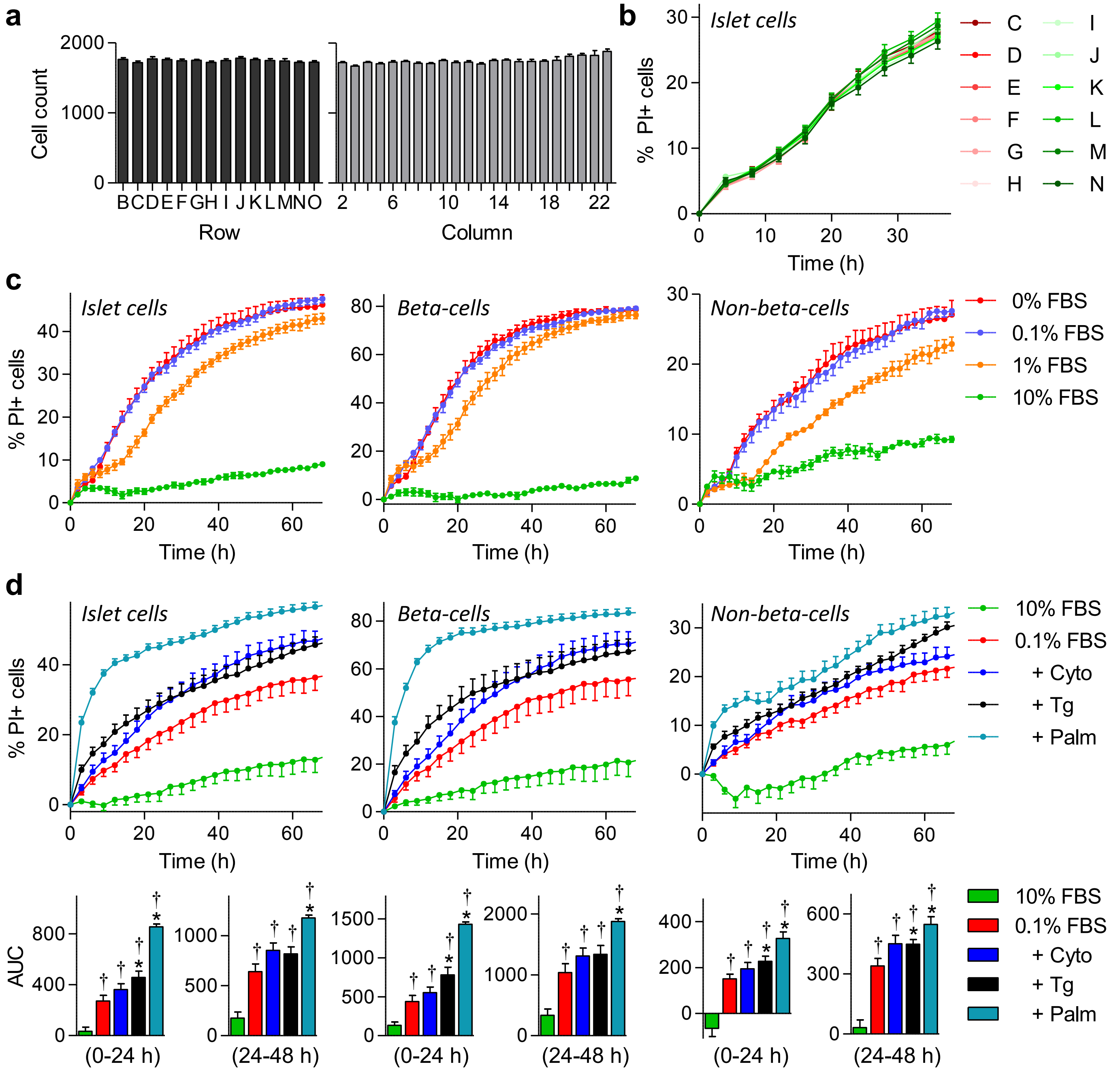

ESM Figure S1. High content screening assays for conducting parallel comparisons of the survival effects of a library of factors. ***a.*** Consistent seeding density of dispersed mouse islet-cells in 384-well plates (n=14,22; mean ± SEM). ***b.*** Islet-cell death assessed by quantifying the PI-positive cell percentage over time with no significant row biases (n=20). ***c.*** Percentage of PI-positive MIP-GFP islet-cells treated with a gradient of FBS under 5 mmol/l glucose (n=4). ***d.*** MIP-GFP islet-cells treated with 0.1% FBS under 5 mmol/l glucose conditions and in the context of cytokines, thapsigargin, or palmitate. 10% FBS was a positive pro-survival control. PI-positive cell percentage (PI^+) was determined and area-under-the-curve for the indicated time intervals calculated (n=16-18, *p<0.05 versus 0.1% FBS; †p<0.05 versus 10% FBS).
