# Supplementary material for: A live-cell, high-content imaging survey of 206 endogenous factors across five stress conditions reveals context-dependent survival effects in mouse primary beta cells
Source: Diabetologia. 2015 Mar 14;58(6):1239–49. doi: 10.1007/s00125-015-3552-5 (PMC4415993; doi:10.1007/s00125-015-3552-5)
Supplement: Supplementary file 3 — (PDF 820 kb) [file 125_2015_3552_MOESM3_ESM.pdf]

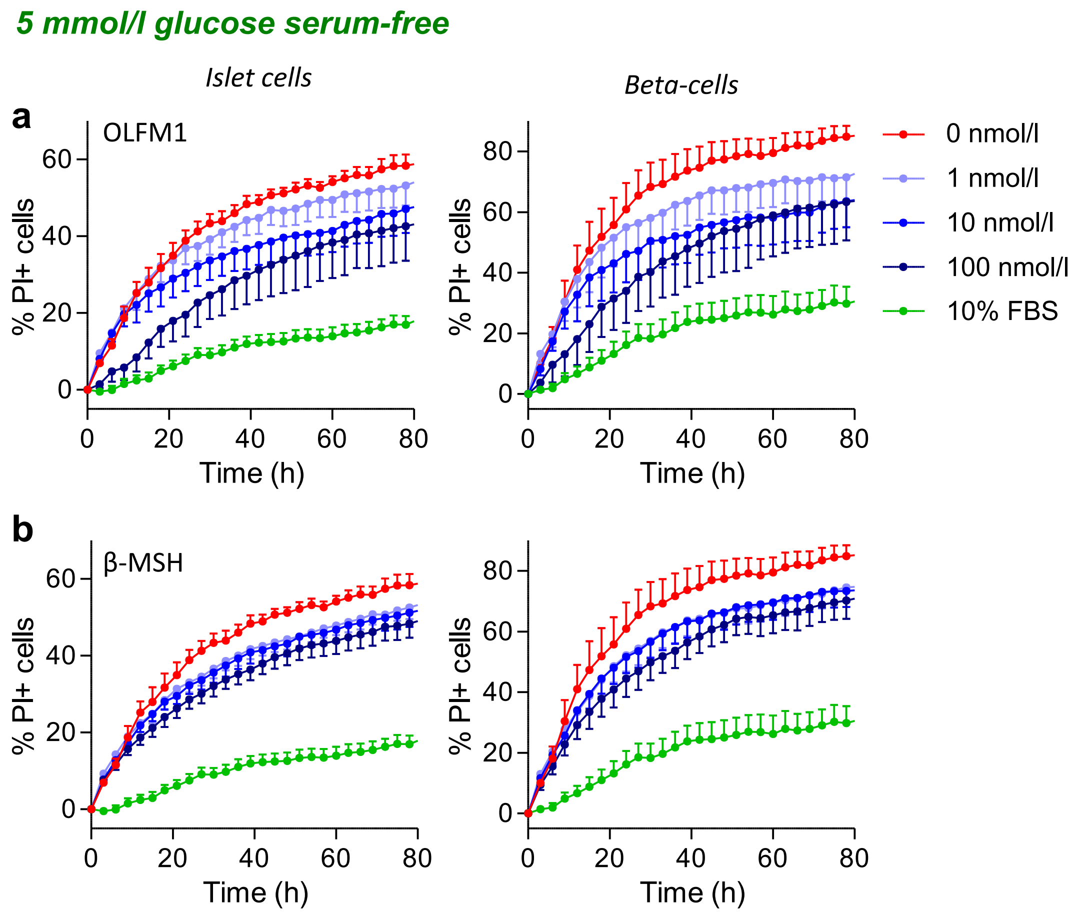

ESM Figure S2. Validation of OLFM1 and beta-MSH on beta cell survival in the context of serum starvation. Dispersed MIP-GFP islet cells were stained and imaged. The percentage of PI^+ cells was determined following treatments with OLFM1 ***(a)*** and β-MSH ***(b)*** at 1, 10, and 100 nmol/l. Cells were concurrently exposed 5 mmol/l glucose serum free stress. 10% FBS was used as positive control for unstressed cells.
