# Supplementary material for: A live-cell, high-content imaging survey of 206 endogenous factors across five stress conditions reveals context-dependent survival effects in mouse primary beta cells
Source: Diabetologia. 2015 Mar 14;58(6):1239–49. doi: 10.1007/s00125-015-3552-5 (PMC4415993; doi:10.1007/s00125-015-3552-5)
Supplement: Supplementary file 7 — (PDF 323 kb) [file 125_2015_3552_MOESM7_ESM.pdf]

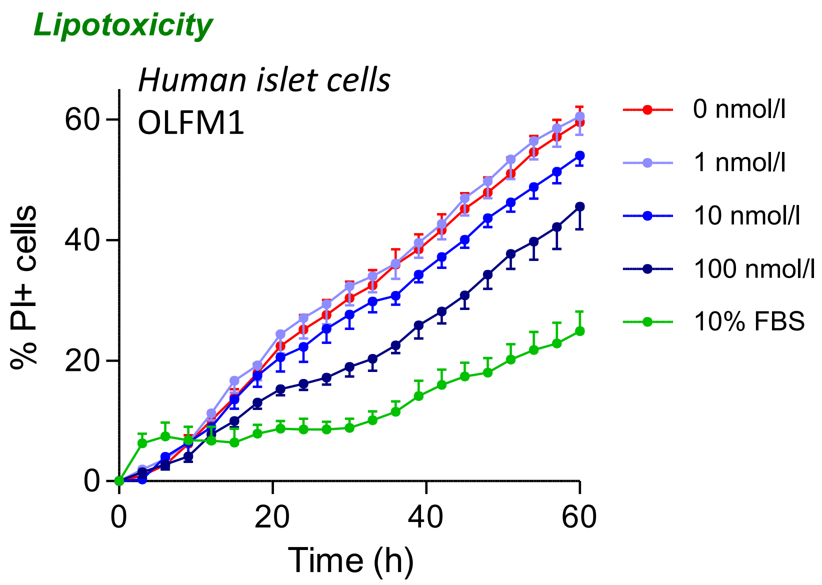

ESM Figure S6. Validation of OLFM1 on islet cell survival in the context of lipotoxicity. Dispersed human islet-cells were stained and imaged. The percentage of PI^{+} cells was determined following treatments with OLFM1 at 1, 10, and 100 nM. Cells were concurrently exposed to palmitate in 5 mmol/l glucose serum free conditions. 10% FBS was used as positive control for unstressed cells.
