# Supplementary material for: A live-cell, high-content imaging survey of 206 endogenous factors across five stress conditions reveals context-dependent survival effects in mouse primary beta cells
Source: Diabetologia. 2015 Mar 14;58(6):1239–49. doi: 10.1007/s00125-015-3552-5 (PMC4415993; doi:10.1007/s00125-015-3552-5)
Supplement: Supplementary file 8 — (PDF 466 kb) [file 125_2015_3552_MOESM8_ESM.pdf]

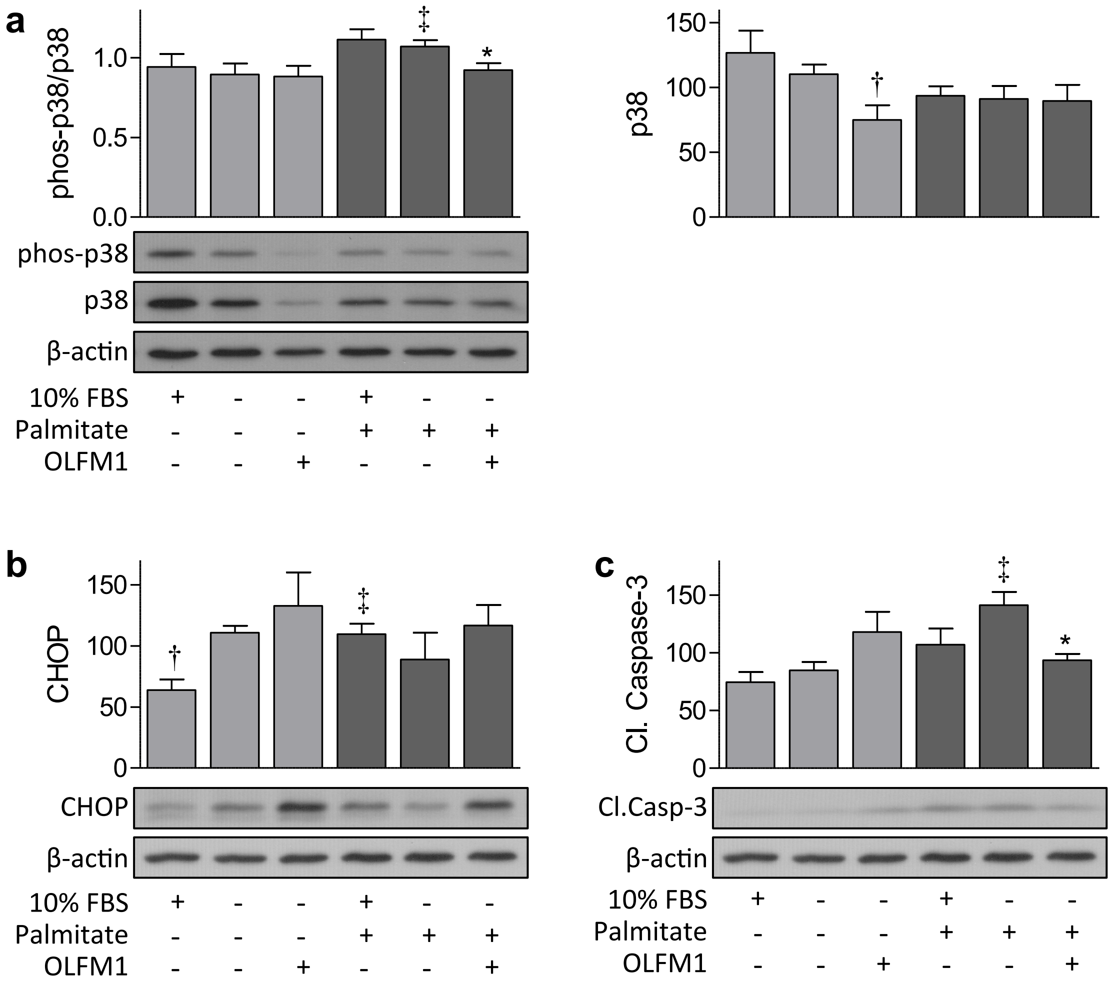

ESM Figure S7. OLFM1 down-regulates stress signalling induced by lipotoxicity in mouse islets. Whole mouse islets were treated for 24 h with 100 nM OLFM1 under the indicated conditions. Western blotting for phospho-p38 MAPK, p38 MAPK (***a*,*b***), CHOP (***c***), and cleaved caspase-3 (***d***) (n=4-8, *p<0.05 versus serum free + Palmitate; ‡p<0.05 versus non-palmitate treated; †p<0.05 versus serum free).
