# Supplementary material for: A live-cell, high-content imaging survey of 206 endogenous factors across five stress conditions reveals context-dependent survival effects in mouse primary beta cells
Source: Diabetologia. 2015 Mar 14;58(6):1239–49. doi: 10.1007/s00125-015-3552-5 (PMC4415993; doi:10.1007/s00125-015-3552-5)
Supplement: Supplementary file 14 — (PDF 651 kb) [file 125_2015_3552_MOESM14_ESM.pdf]

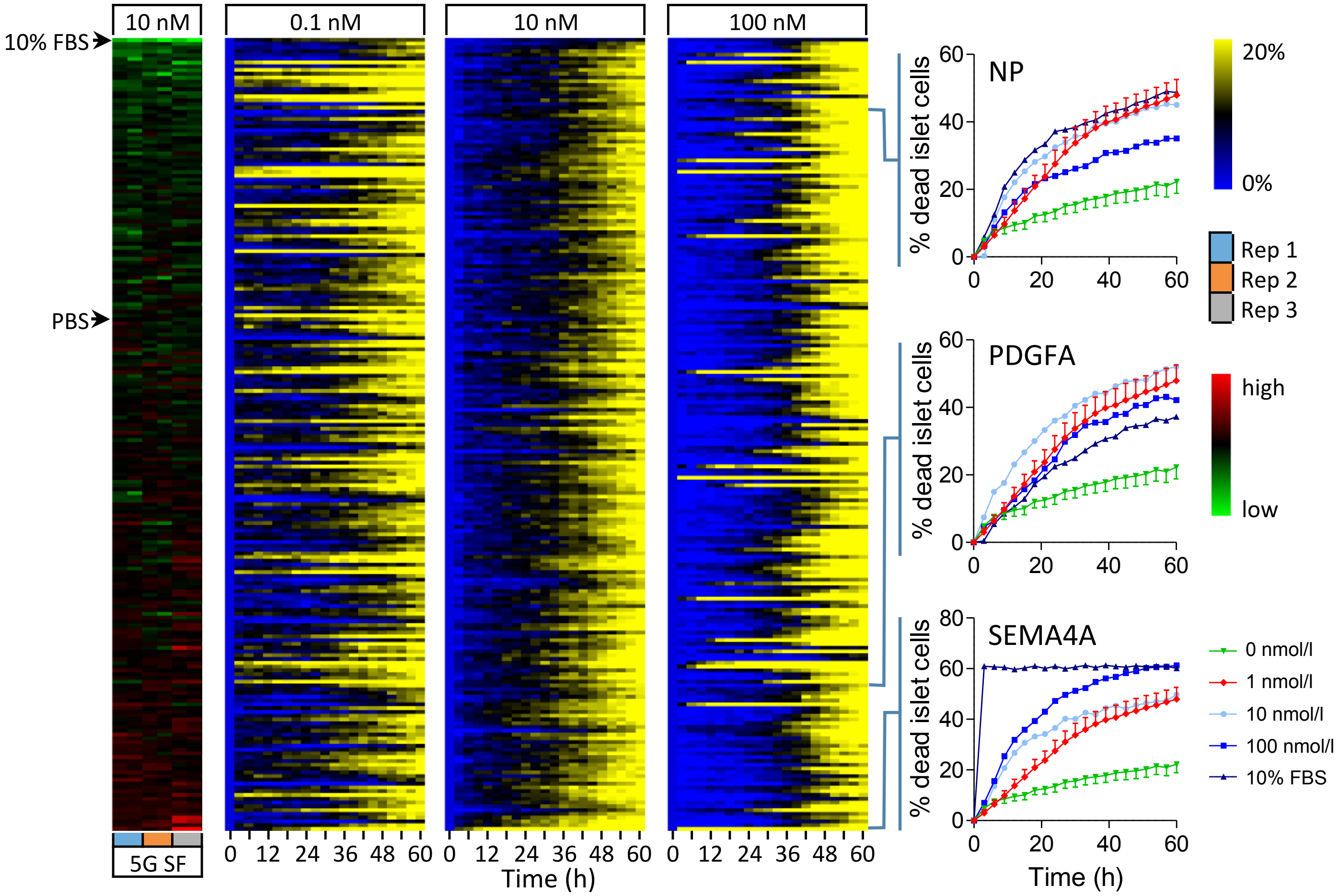

ESM Figure S13. Multiple factors display concentration dependent transient or persistent protective effects. Dispersed mouse islet cells were stained and imaged. The percentage of PI^{+} cells was determined following treatments with a library of 206 factors at 0.1, 10, and 100 nM each. Cells were concurrently exposed 5 mM glucose serum free stress. 10% FBS was used as positive control for unstressed cells. *Left panel:* In the green to red heat map, data are presented as z-scores for the 0-24 hand 24-48 h time intervals for each replicate experiment and the factors were ranked for their protective effects (low levels of PI^{+} cells equates to high protection). *Middle panel:* In the blue to yellow heat map, data are presented as $\%PI^{+}$ cells at each timepoint. *Right panel:* Examples of factors showing concentration dependent effects on cell survival.
