# Supplementary material for: A live-cell, high-content imaging survey of 206 endogenous factors across five stress conditions reveals context-dependent survival effects in mouse primary beta cells
Source: Diabetologia. 2015 Mar 14;58(6):1239–49. doi: 10.1007/s00125-015-3552-5 (PMC4415993; doi:10.1007/s00125-015-3552-5)
Supplement: Supplementary file 15 — (PDF 229 kb) [file 125_2015_3552_MOESM15_ESM.pdf]

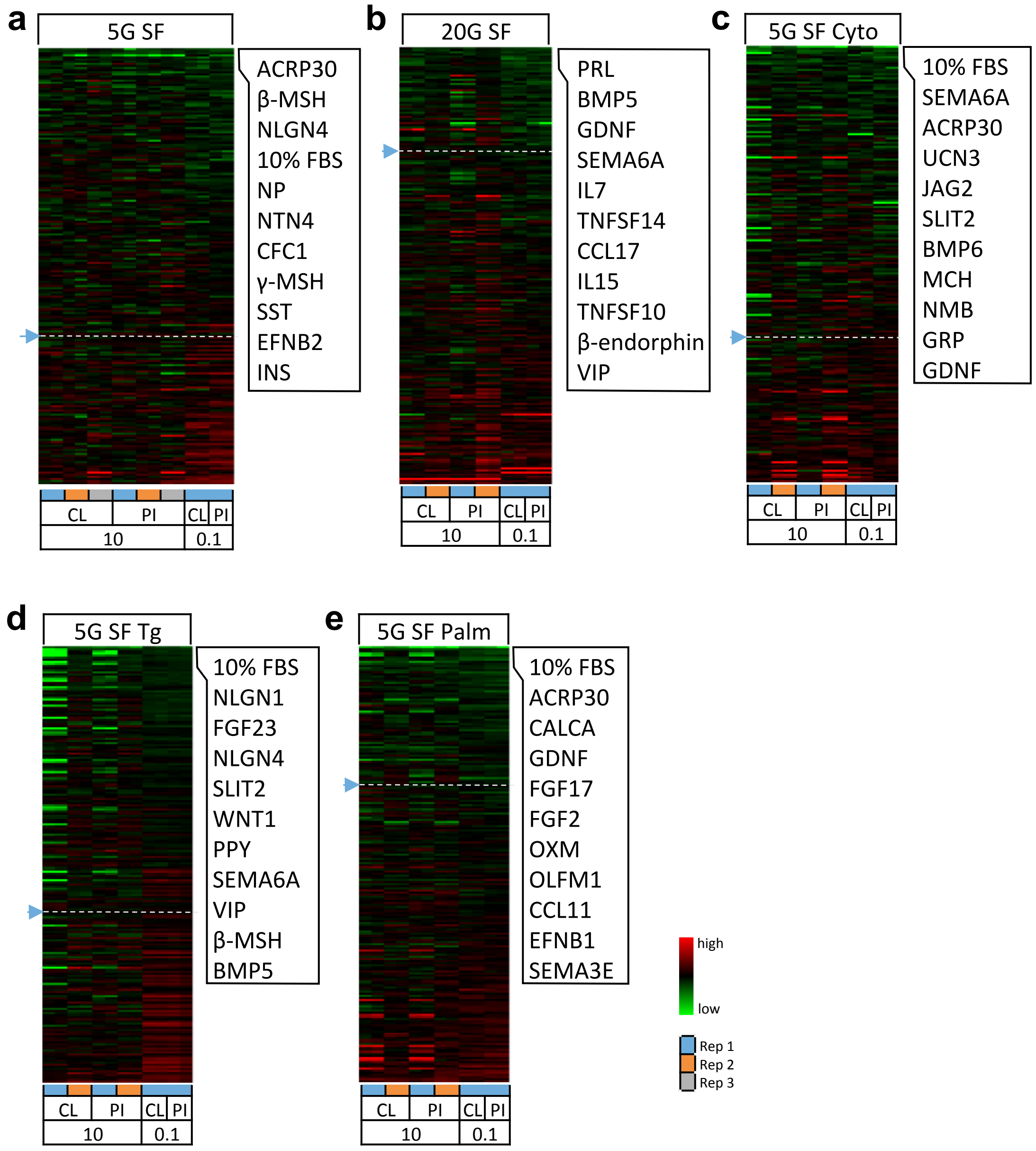

ESM Figure S14. Multiple factors display concentration dependent stress specific protective effects. A-E. Dispersed mouse islet cells were stained and imaged. The level of cell loss and percentage of PI^+ cells was determined following treatments with a library of 206 factors at 10 nM each. Cells were concurrently exposed to one of five stress conditions, including 20 mM glucose serum-free (SF), and 5 mM glucose SF only and in combination with a cytokine cocktail (25 ng/ml TNF-α, 10 ng/ml IL-1β, 10 ng/ml IFN-γ), 1 µM thapsigargin, and/or 1.5 mM palmitate. 10% FBS was used as positive control for unstressed cells. Data are presented as z-scores for the 0-24 hand 24-48 h time intervals for each replicate experiment. The factors were ranked for their protective effects based on low levels of cell loss and low levels of PI^+ cells under the stress treatments indicated at the top of each heat map. The top 10 most protective factors under each condition are listed in the callout.
