# Supplementary material for: A live-cell, high-content imaging survey of 206 endogenous factors across five stress conditions reveals context-dependent survival effects in mouse primary beta cells
Source: Diabetologia. 2015 Mar 14;58(6):1239–49. doi: 10.1007/s00125-015-3552-5 (PMC4415993; doi:10.1007/s00125-015-3552-5)
Supplement: Supplementary file 16 — (PDF 288 kb) [file 125_2015_3552_MOESM16_ESM.pdf]

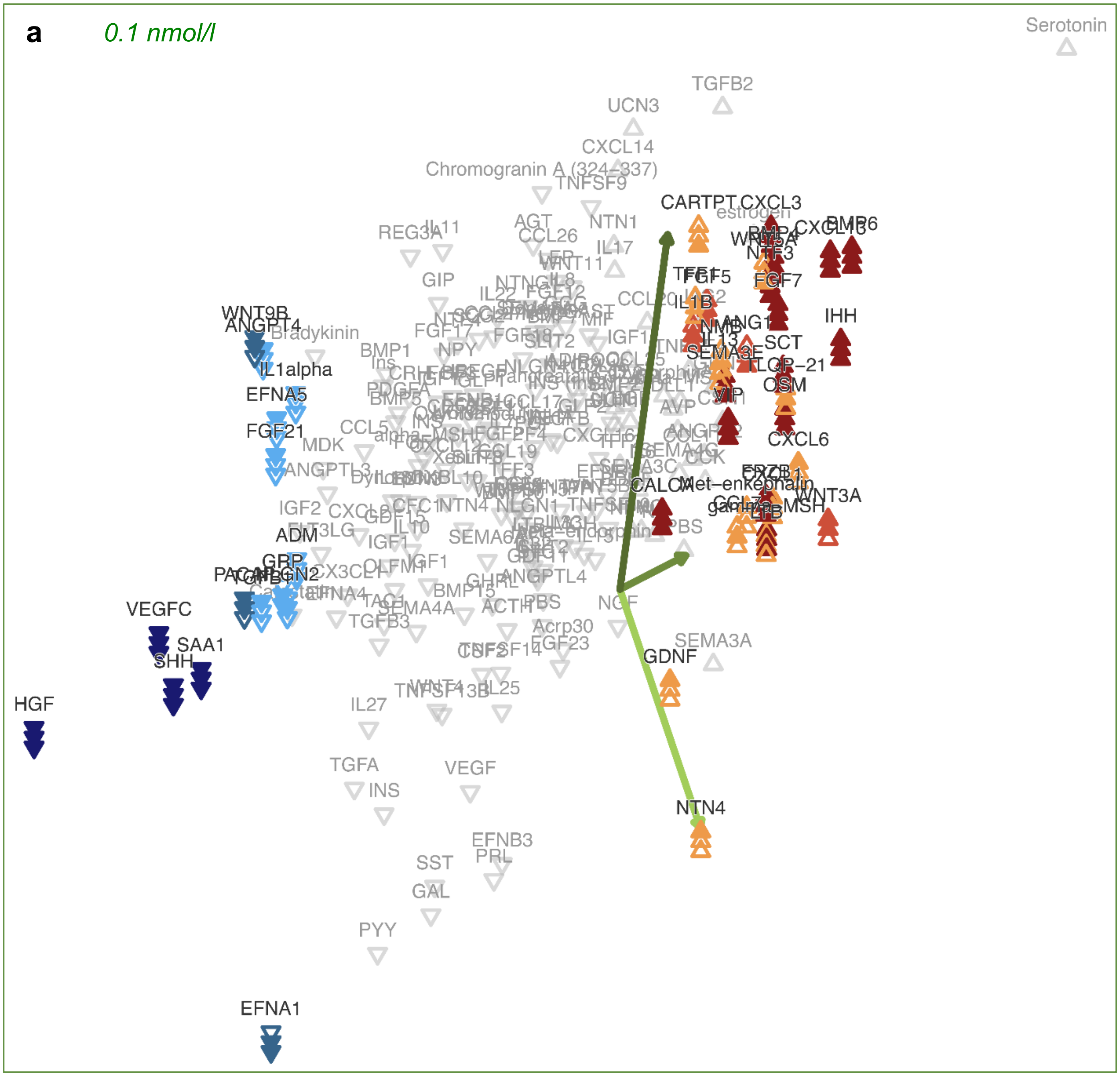

ESM Figure S15. Rank product test analysis of 0.1 nmol/l of each factor across all tested conditions. Rank product test statistical analysis of PI^{+} cell data only, described in the Methods, represented in a PCA plot. Factors that are nominally significant for any one day are highlighted with three arrows (representing three days: top arrow = day 1, middle arrow = day 2, and bottom arrow = day 3). An arrow that is coloured is significant for that day.
