# Supplementary material for: A live-cell, high-content imaging survey of 206 endogenous factors across five stress conditions reveals context-dependent survival effects in mouse primary beta cells
Source: Diabetologia. 2015 Mar 14;58(6):1239–49. doi: 10.1007/s00125-015-3552-5 (PMC4415993; doi:10.1007/s00125-015-3552-5)
Supplement: Supplementary file 17 — (PDF 107 kb) [file 125_2015_3552_MOESM17_ESM.pdf]

ESM Table S1. List of recombinant biologic factors

Gene Symbol	Protein/Peptide Name	Manufacturer *	Cat. No.	Source
ACRP30	adiponectin (NS0 cells-derived)	R&D Systems	1065-AP	H
ACRP30	adiponectin (Hi-5 insect cells-derived, ADIPOQ)	Peprotech	450-24	H
ADM	adrenomedullin 52	Sigma	A2327	H
AGT	angiotensinogen, Angiothesin II	Sigma	A9525	H
ANG1	angiopoietin 1	Peprotech	130-06	H
ANGPT2	angiopoietin 2	R&D Systems	623-AN-025	H
ANGPT4	angiopoietin 4	R&D Systems	964-AN-025	H
ANGPTL3	angiopoietin-like 3	R&D Systems	3829-AN-050	H
ANGPTL4	angiopoietin-like 4	R&D Systems	4487-AN-050	H
AVP	arginine vasopressin	Tocris	2935	H
BDNF	brain-derived neurotrophic factor	Peprotech	450-02	H
BMP1	bone morphogenetic protein 1	R&D Systems	1927-ZN-010	H
BMP10	bone morphogenetic protein 10	R&D Systems	2926-BP-025	H
BMP15	bone morphogenetic protein 15 (GDF9B)	R&D Systems	5096-BM-005	H
BMP2	bone morphogenetic protein 2	Peprotech	120-02	H
BMP4	bone morphogenetic protein 4	Peprotech	120-05	H
BMP5	bone morphogenetic protein 5	R&D Systems	615-BM-050	H
BMP6	bone morphogenetic protein 6	Peprotech	120-06	H
BMP7	bone morphogenetic protein 7 (osteogenic protein 1)	Peprotech	120-03	H
BTC	betacellulin	Peprotech	100-50	H
CALCA	calcitonin-related polypeptide alpha	Sigma	C0167	H
CARTPT	cocaine and amphetamine regulated transcript peptide (55-102)	Sigma	C5977	H
CCK	cholecystokinin (CCK Octapeptide)	Tocris	1166	H
CCL11	chemokine (C-C motif) ligand 11 (eotaxin)	Peprotech	300-21	H
CCL16	chemokine (C-C motif) ligand 16 (LEC)	Peprotech	300-44	H
CCL17	chemokine (C-C motif) ligand 17 (TARC)	Peprotech	300-30	H
CCL19	chemokine (C-C motif) ligand 19 (MIP-3 beta)	Peprotech	300-29B	H
CCL2	chemokine (C-C motif) ligand 2 (MCP-1)	Peprotech	300-04	H
CCL20	chemokine (C-C motif) ligand 20 (MIP-3 alpha)	Peprotech	300-29A	H
CCL25	chemokine (C-C motif) ligand 25 (TECK)	Peprotech	300-45	H
CCL26	chemokine (C-C motif) ligand 26 (Eotaxin-3)	Peprotech	300-48	H
CCL27	chemokine (C-C motif) ligand 27 (CTACK)	Peprotech	300-54	H

Gene Symbol	Protein/Peptide Name	Manufacturer *	Cat. No.	Source
CCL4	chemokine (C-C motif) ligand 4 (MIP-1 beta)	Peprotech	300-09	H
CCL5	chemokine (C-C motif) ligand 5 (RANTES)	Peprotech	300-06	H
CCL7	chemokine (C-C motif) ligand 7 (MCP-3)	Peprotech	300-17	H
CD55	decay accelerating factor (DAF)	R&D Systems	2009-CD-050	H
CFC1	cripto, FRL-1, cryptic family 1 (cryptic)	R&D Systems	1410-CR-050	H
CHGA	pancreastatin (37-52)	Sigma	P9809-.5MG	H
CHGA	WE 14 (324-337)	Sigma	SCP0116-1MG	H
CHGA	catestatin	Sigma	C6249-1MG	H
COPA	xenin-8	Tocris	2138	H
CRH	corticotropin releasing hormone	Sigma	C3042	H
CSF1	colony stimulating factor 1 (M-CSF)	Peprotech	300-25	H
CSF2	colony stimulating factor 2 (GM-CSF)	Peprotech	300-03	H
CSH1	placental lactogen	R&D Systems	5757-PL-025	H
CX3CL1	chemokine (C-X3-C motif) ligand 1 (Fractalkine)	Peprotech	300-31	H
CXCL1	chemokine (C-X-C motif) ligand 1 (GRO alpha)	Peprotech	300-11	H
CXCL10	chemokine (C-X-C motif) ligand 10 (IP-10)	Peprotech	300-12	H
CXCL11	chemokine (C-X-C motif) ligand 11 (I-TAC)	Peprotech	300-46	H
CXCL12	chemokine (C-X-C motif) ligand 12 (SDF-1 alpha)	Peprotech	300-28A	H
CXCL13	chemokine (C-X-C motif) ligand 13 (BCA-1)	Peprotech	300-47	H
CXCL14	chemokine (C-X-C motif) ligand 14 (BRAK)	Peprotech	300-50	H
CXCL16	chemokine (C-X-C motif) ligand 16	Peprotech	300-55	H
CXCL2	chemokine (C-X-C motif) ligand 2 (GRO beta)	Peprotech	300-39	H
CXCL3	chemokine (C-X-C motif) ligand 3 (GRO gamma)	Peprotech	300-40	H
CXCL6	chemokine (C-X-C motif) ligand 6 (GCP-2)	Peprotech	300-41	H
DLL1	delta-like 1 (Drosophila)	Peprotech	140-08	H
EDN3	endothelin 3	Sigma	E9137	H
EFNA1	ephrin-A1	R&D Systems	602-A1-200	M
EFNA4	ephrin-A4	R&D Systems	369-EA-200	H
EFNA5	ephrin-A5	R&D Systems	374-EA-200	H
EFNB1	ephrin-B1	R&D Systems	473-EB-200	M
EFNB2	ephrin-B2	R&D Systems	496-EB-200	M
EFNB3	ephrin-B3	Sigma	E0903	H
EGF	epidermal growth factor	Peprotech	AF-100-15	H

Gene Symbol	Protein/Peptide Name	Manufacturer *	Cat. No.	Source
estrogen	estrogen (estradiol, alpha)	Tocris	2823	H
FGF1	fibroblast growth factor 1 (acidic)	Peprotech	100-17A	H
FGF12	fibroblast growth factor 12	R&D Systems	2246-FG-025	H
FGF17	fibroblast growth factor 17	Peprotech	100-27	H
FGF18	fibroblast growth factor 18	Peprotech	100-28	H
FGF2	fibroblast growth factor 2 (basic)	Peprotech	100-18B	H
FGF21	fibroblast growth factor 21	Peprotech	100-42	H
FGF23	fibroblast growth factor 23	Peprotech	100-52	H
FGF3	fibroblast growth factor 3	R&D Systems	1206-F3-025	H
FGF5	fibroblast growth factor 5	Peprotech	100-34	H
FGF7	fibroblast growth factor 7 (keratinocyte growth factor)	Peprotech	100-19	H
FGF9	fibroblast growth factor 9 (glia-activating factor)	Peprotech	100-23	H
FLT3LG	fms-related tyrosine kinase 3 ligand	Peprotech	300-19	H
FRZB	frizzled-related protein (sFRP-3)	R&D Systems	192-SF-010	H
GAL	galanin (1-30)	Tocris	1179	H
GAST	gastrin	Sigma	G9020	H
GCG	glucagon	Sigma	G2044-1mg	H
GCG	oxyntomodulin (OXM)	Tocris	2094	H
GCG	glucagon-like peptide 1 (GLP-1)	Peprotech	130-08	H
GCG	glucagon-like peptide 2 (GLP-2)	Tocris	2258	H
GDF11	growth differentiation factor 11 (BMP11)	Peprotech	120-11	H
GDF15	growth differentiation factor 15	Peprotech	120-28	H
GDNF	glial cell line-derived neurotrophic factor	Peprotech	450-10	H
GHRL	ghrelin	Tocris	1463	H
GIP	gastric inhibitory polypeptide	Tocris	2257	H
GRN	progranulin	R&D Systems	2420-PG-050	H
GRP	gastrin-releasing peptide	Tocris	1789	H
HBEGF	heparin-binding EGF-like growth factor	Peprotech	100-47	H
HGF	hepatocyte growth factor (scatter factor)	Peprotech	100-39	H
IAPP	islet amyloid polypeptide (amylin)	Tocris	3418	H
IFNG	interferon gamma	Peprotech	300-02	H
IGF1	insulin like growth factor-1	R&D systems	291-G1	H
IGF2	insulin-like growth factor 2 (somatomedin A)	Peprotech	100-12	H

Gene Symbol	Protein/Peptide Name	Manufacturer *	Cat. No.	Source
IHH	Indian hedgehog homolog (Drosophila)	R&D Systems	1705-HH-025	M
IL10	interleukin 10	Peprotech	200-10	H
IL11	interleukin 11	Peprotech	200-11	H
IL13	interleukin 13	Peprotech	200-13	H
IL15	interleukin 15	Peprotech	200-15	H
IL17	interleukin 17	Peprotech	200-17	H
IL18	interleukin 18 (interferon-gamma-inducing factor)	R&D Systems	B001-5	H
IL1alpha	interleukin 1alpha	Peprotech	200-01A	H
IL1beta	interleukin 1beta	Peprotech	200-01B	H
IL22	interleukin 22	Peprotech	200-22	H
IL25	interleukin 25 (IL-17E)	Peprotech	200-24	H
IL27	interleukin 27 (IL-17D)	Peprotech	200-27	H
IL32	interleukin 32 (IL-32 gamma)	R&D Systems	4690-IL-025/CF	H
IL33	interleukin 33	Peprotech	200-33	H
IL4	interleukin 4	Peprotech	200-04	H
IL6	interleukin 6	Peprotech	200-06	H
IL7	interleukin 7	Peprotech	200-07	H
IL8	interleukin 8 (CXCL8)	Peprotech	200-08M	H
INHA	inhibin, alpha (Inhibin-Like Peptide, human)	Sigma	I9638	H
INHBA	inhibin, beta 1 Activin A (Activin A)	Peprotech	120-14E	H
INS	insulin	Sigma	I-9278	H
JAG2	jagged 2	R&D Systems	1726-JG-050	H
KNG1	bradykinin	Tocris	3004	H
LEP	leptin	Peprotech	300-27	H
LTB	lymphotoxin alpha2/beta1	R&D Systems	679-TX-010	H
MCH	melanin-concentrating hormone	Tocris	3434	H
MDK	midkine (neurite growth-promoting factor 2)	Peprotech	450-16	H
MIF	macrophage migration inhibitory factor	R&D Systems	289-MF-002	H
NGF	nerve growth factor (beta polypeptide)	Peprotech	450-01	H
NLGN1	neuroligin 1	R&D Systems	4340-NL-050	R
NLGN2	neuroligin 2	R&D Systems	5645-NL-050	H
NLGN4	neuroligin 4	R&D Systems	5158-NL-050	H
NMB	neuromedin B	Tocris	1908	H

Gene Symbol	Protein/Peptide Name	Manufacturer *	Cat. No.	Source
NP	natriuretic peptide (atrial natriuretic factor 1-28)	Tocris	1912	R
NPW	neuropeptide W-23	Tocris	3009	H
NPY	neuropeptide Y	Sigma	N5017	H
NTF3	neurotrophin-3 (NT-3, NGF2)	Peprotech	450-03	H
NTF4	neurotrophin-4/5 (NT-4/NT-5)	Peprotech	450-04	H
NTN1	netrin 1	R&D Systems	1109-N1-025	M
NTN4	netrin 4	R&D Systems	1254-N4-025	H
NTN4	netrin 4	R&D Systems	1132-N4-025/CF	M
NTNG1	netrin G1-a	R&D Systems	1166-NG-025	M
OLFM1	olfactomedin 1 (noelin-1)	R&D Systems	4636-NL-050	H
OSM	oncostatin M	Peprotech	300-10	H
PACAP	pituitary adenylate cyclase activating polypeptide (1-38)	Tocris	1186	H
PDGFA	platelet-derived growth factor alpha polypeptide	Peprotech	100-13A	H
PF4	platelet factor 4 (chemokine (C-X-C motif) ligand 4)	Peprotech	300-16	H
PGF	placental growth factor (PIGF)	Peprotech	100-06	H
PNOC	[Phe1-ψ(CH2-NH)-Gly2]-Nociceptin Fragment 1-13 amide	Sigma	N9908	H
POMC	α-melanocyte stimulating hormone (α-MSH)	Sigma	M4135	H
POMC	β-melanocyte stimulating hormone (β-MSH)	Sigma	M6513	H
POMC	γ-melanocyte stimulating hormone (γ-MSH)	Sigma	M9638	H
POMC	adrenocorticotropic hormone (ACTH)	Sigma	A0423	H
POMC	Met-enkephalin (M-ENK)	Tocris	1889	H
POMC	β-endorphin	Sigma	E6261	H
PPY	pancreatic polypeptide	Sigma	P9903	H
PRL	prolactin	Peprotech	100-07	H
PYDN	dynorphinA	Tocris	3195	H
PYDN	dynorphinB	Tocris	3196	H
PYY	peptide YY	Sigma	P1306	H
REG3A	regenerating islet-derived 3 alpha	R&D Systems	3907-RG-050	M
SAA1	serum amyloid A1 (Apo-SAA1)	Peprotech	300-53	H
SCG2	secretogranin II (chromogranin C, secretoneurin)	Sigma	S7065	M
SCT	secretin	Tocris	1918	H
SEMA3A	semaphorin 3A	R&D Systems	1250-S3-025	H
SEMA3C	semaphorin 3C	R&D Systems	5570-S3-050	H

Gene Symbol	Protein/Peptide Name	Manufacturer *	Cat. No.	Source
SEMA3E	semaphorin 3E	R&D Systems	3239-S3-025	H
SEMA4A	semaphorin 4A	R&D Systems	4694-S4-050	H
SEMA4G	semaphorin 4G	R&D Systems	5840-S4-050	H
SEMA5A	semaphorin 5A	R&D Systems	5896-S5-025	H
SEMA6A	semaphorin 6A	R&D Systems	1146-S6-025	H
SHH	sonic hedgehog	Peprotech	100-45	H
SLIT1	slit homolog 1 (Drosophila)	R&D Systems	5199-SL-050	H
SLIT2	slit homolog 2 (Drosophila)	Peprotech	150-11	H
SLIT2	slit homolog 2 (Drosophila)	R&D Systems	5444-SL-050	M
SLIT3	slit homolog 3 (Drosophila)	R&D Systems	3629-SL-050	H
SST	somatostatin-14	Tocris	1157	H
TAC1	tachykinin, precursor 1 (α-neurokinin fragment 4-10)	Sigma	N5141	H
TFF1	trefoil factor 1	Peprotech	300-60	H
TFF2	trefoil factor 2 (spasmolytic protein 1)	Peprotech	300-59	H
TFF3	trefoil factor 3 (intestinal)	Peprotech	300-61	H
TGFA	transforming growth factor, alpha	Peprotech	100-16A	H
TGFB1	transforming growth factor, beta 1	Peprotech	100-21C	H
TGFB2	transforming growth factor, beta 2	Peprotech	100-35	H
TGFB3	transforming growth factor, beta 3	Peprotech	100-36E	H
TNFSF10	tumor necrosis factor (ligand) superfamily, member 10 (TRAIL)	Peprotech	310-04	H
TNFSF13B	tumor necrosis factor (ligand) superfamily, member 13b (BAFF)	Peprotech	310-13	H
TNFSF14	tumor necrosis factor (ligand) superfamily, member 14 (LIGHT)	Peprotech	310-09B	H
TNFSF15	tumor necrosis factor (ligand) superfamily, member 15 (TL-1A)	Peprotech	310-23	H
TNFSF4	tumor necrosis factor (ligand) superfamily, member 4 (OX40L)	Peprotech	310-28	H
TNFSF9	tumor necrosis factor (ligand) superfamily, member 9 (4-IBBL)	Peprotech	310-11	H
UCN	urocortin	Sigma	U4127	H
UCN2	urocortin 2	Sigma	S5441	H
UCN3	urocortin 3	Sigma	U1008	H
VEGF	vascular endothelial growth factor	Peprotech	100-20	H
VEGFB	vascular endothelial growth factor B	Peprotech	100-20B	H
VEGFC	vascular endothelial growth factor C	Peprotech	100-20C	H
VGF	TLQP-21	Tocris	3051	H
VIP	vasoactive intestinal peptide	Sigma	V3628	H

Gene Symbol	Protein/Peptide Name	Manufacturer *	Cat. No.	Source
WNT1	wingless-type MMTV integration site family, member 1	Peprotech	120-17	H
WNT11	wingless-type MMTV integration site family, member 11	R&D Systems	6179-WN-010/CF	H
WNT3A	wingless-type MMTV integration site family, member 3A	Peprotech	120-27	H
WNT4	wingless-type MMTV integration site family, member 4	R&D Systems	475-WN-005	H
WNT5A	wingless-type MMTV integration site family, member 5A	R&D Systems	645-WN-010	H
WNT5B	wingless-type MMTV integration site family, member 5B	R&D Systems	3006-WN-025	H
WNT7A	wingless-type MMTV integration site family, member 7A	Peprotech	120-31	H
WNT9B	wingless-type MMTV integration site family, member 9B	R&D Systems	3669-WN-025	H
	serotonin (5-HT)	Tocris	3547	H
	dopamine	Tocris	3548	H

***** Manufacturers: R&D Systems (Minneapolis, MN, USA), Peprotech (Rocky Hill, NJ, USA), Sigma (St Louis, MO, USA), Tocris (Avonmouth, Bristol, UK)
