# Supplementary material for: A live-cell, high-content imaging survey of 206 endogenous factors across five stress conditions reveals context-dependent survival effects in mouse primary beta cells
Source: Diabetologia. 2015 Mar 14;58(6):1239–49. doi: 10.1007/s00125-015-3552-5 (PMC4415993; doi:10.1007/s00125-015-3552-5)
Supplement: Supplementary file 12 — (PDF 296 kb) [file 125_2015_3552_MOESM12_ESM.pdf]

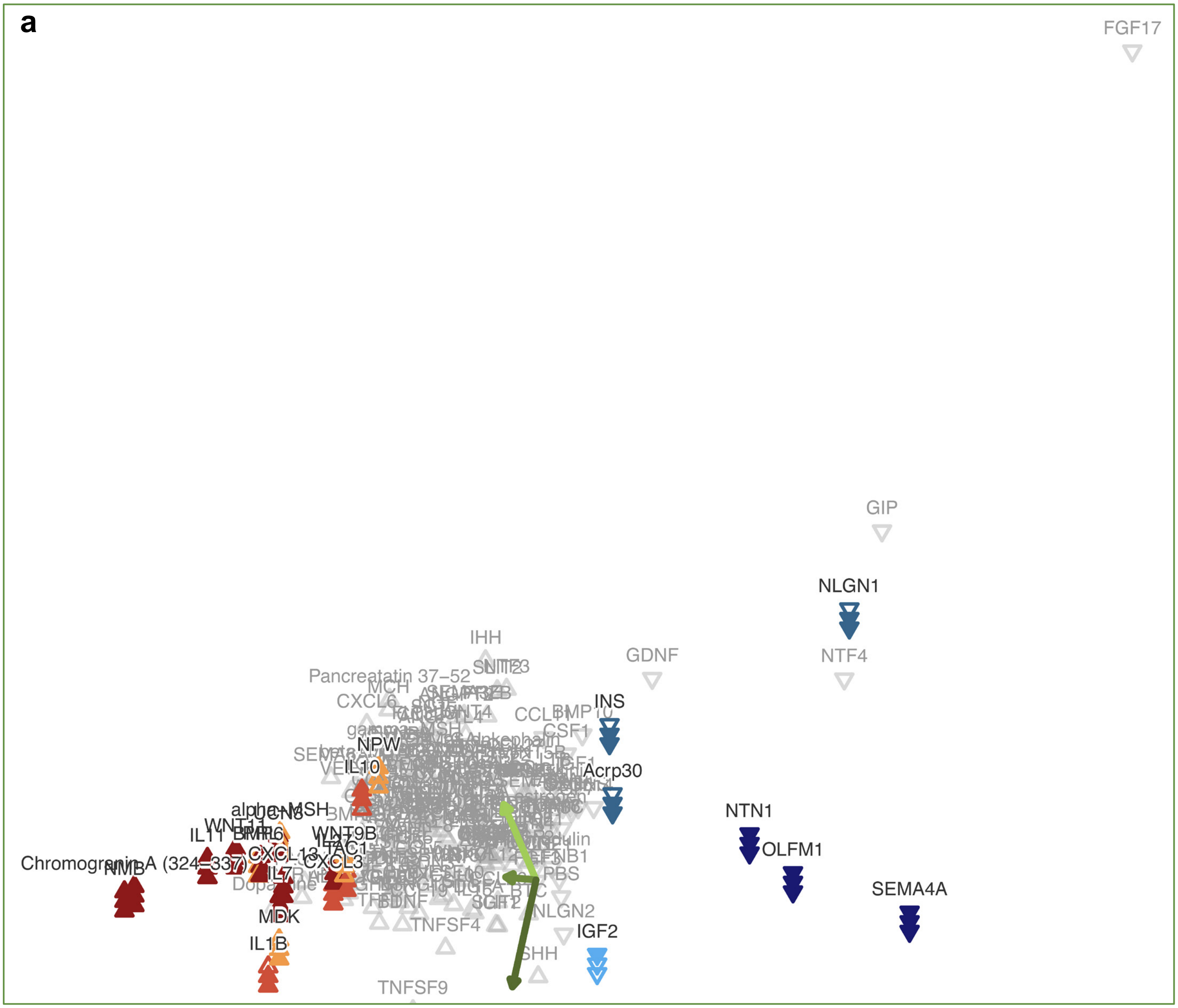

ESM Figure S11. Principal component analysis plot of rank product testing of 206 factors in a lipotoxic condition. ***a.*** Rank product analysis of PI^{+} cell data. The first component in the PCA plot illustrates the agreement between the days, whereas the second component highlights their differences. Nominally significant factors for any one day are highlighted with three arrows (representing three days: top arrow = day 1, middle arrow = day 2, and bottom arrow = day 3). Colored arrows are significant for that day. This Supplemental Figure accompanies Figure 5.
